# The rapid generation of chimerical genes expanding protein diversity in zebrafish

**DOI:** 10.1186/1471-2164-11-657

**Published:** 2010-11-24

**Authors:** Beide Fu, Ming Chen, Ming Zou, Manyuan Long, Shunping He

**Affiliations:** 1 The Key Laboratory of Aquatic Biodiversity and Conservation of Chinese Academy of Sciences, Institute of Hydrobiology, Chinese Academy of Sciences, Wuhan 430072, PR China; 2 Graduate University of the Chinese Academy of Sciences, Beijing 100039, PR China; 3 Department of Ecology and Evolution, University of Chicago, Chicago 60637, Illinois

## Abstract

**Background:**

Variation of gene number among species indicates that there is a general process of new gene origination. One of the major mechanism providing raw materials for the origin of new genes is gene duplication. Retroposition, as a special type of gene duplication- the RNA-based duplication, has been found to play an important role in new gene evolution in mammals and plants, but little is known about the process in the teleostei genome.

**Results:**

Here we screened the zebrafish genome for identification of retrocopies and new chimerical retrogenes and investigated their origination and evolution. We identified 652 retrocopies, of which 440 are intact retrogenes and 212 are pseudogenes. Retrocopies have long been considered evolutionary dead ends without functional significance due to the presumption that retrocopies lack the regulatory element needed for expression. However, 437 transcribed retrocopies were identified from all of the retrocopies. This discovery combined with the substitution analysis suggested that the majority of all retrocopies are subject to negative selection, indicating that most of the retrocopies may be functional retrogenes. Moreover, we found that 95 chimerical retrogenes had recruited new sequences from neighboring genomic regions that formed *de novo *splice sites, thus generating new intron-containing chimeric genes. Based on our analysis of 38 pairs of orthologs between *Cyprinus carpio *and *Danio rerio*, we found that the synonymous substitution rate of zebrafish genes is 4.13×10^-9 ^substitution per silent site per year. We also found 10 chimerical retrogenes that were created in the last 10 million years, which is 7.14 times the rate of 0.14 chimerical retrogenes per million years in the primate lineage toward human and 6.25 times the rate of 0.16 chimerical genes per million years in *Drosophila*. This is among the most rapid rates of generation of chimerical genes, just next to the rice.

**Conclusion:**

There is compelling evidence that much of the extensive transcriptional activity of retrogenes does not represent transcriptional "noise" but indicates the functionality of these retrogenes. Our results indicate that retroposition created a large amount of new genes in the zebrafish genome, which has contributed significantly to the evolution of the fish genome.

## Background

Retroposition entails a process in which RNA (including mRNA transcribed from a parent gene) is subsequently reverse-transcribed into cDNA and inserted into a new locus on the chromosome to form a new retrocopy locus. The main characteristic of the retrocopy is the lack of introns, and if the retroposition event is recent enough, a poly(A) tail and short flanking duplicate sequences can be observed as well[1]. Retrocopies often become processed pseudogenes (retropseudogenes) and are eventually deleted because they do not have the regulatory elements necessary for expression. However, the extensive structural changes in retrocopies have been speculated as "evolutionary seeds" for the evolution of new gene functions if they acquire new regulatory sequences [2] (hereafter called retrogenes). For example, the finding of the *jingwei *gene in *Drosophila yakuba *and the *sphinx *gene in *Drosophila melanogaster *revealed that the retrogene can recruit a certain regulatory sequence and evolve a new function defined by new expression and new gene structure [3-5]. In the human genome, chimeric retrogenes may originate from small nuclear RNAs and mRNAs by RNA-RNA recombination [6-10]. The structures of such functional retrogenes are usually chimerical: they may either recruit a new regulatory element from the insertion site to form a peculiar coding region that evolved from difference sources [5,11], or they can use exons from genes near the insertion site to form a new gene structure [12,13]. Such chimerical structures usually confer a new function or a new expression pattern that parental genes do not have, thus often leading to adaptive evolution [2,14]. For example, retrogenes in the *Drosophila *genome show a pattern that escaped from the × chromosome to the autochromosome to evolve new testis functions [11,15]. The e(y)2 retrogene in *Drosophila melanogaster *performs a general function and is ubiquitously expressed, while the source gene e(y)2b is functional only in a small group of male germ cells [16]. These studies support the hypothesis that retrogenes played an important role in the evolution of functionality in organisms.

Large numbers of retrogenes have been found in mammal, plant and insect genomes, but few retrogenes have been identified and studied in fish genomes [11,15,17-20]. The first retrogene that was reported in the fish genome was an ocular rod opsin gene, *rho*, which does not have introns, unlike the other intron-containing rod opsin genes in vertebrates, *errlo*, [21]. Previous studies revealed that in the mammal genome, the enzymatic machinery of LINE1 (Long Interspersed Nuclear Element 1, L1) is responsible for the creation of retrogenes [22]. L1 s are widely present in mammals and account for up to about 25% of the genome [23,24]. With the sequenced zebrafish genome, many lineages of L1 s were identified [24,25], therefore we predict that there may be a large amount of retrogenes in the zebrafish genome. Recently, a significant number of transcribed and functional retrocopies were discovered in the primate and rodent genome [11,15,26-29], but there has not been any study of the expressed retrocopies in the teleostei genome.

To understand the functional and evolutionary impact of retroposition in the zebrafish genome, we screened the full genome of zebrafish for retrocopies and chimerical retrogenes. We found that there are 652 retrocopies and 95 chimerical retrogenes in zebrafish genome. This is the first study conducted for retrocopies and chimerical retrogenes in teleostei genome at the whole-genome level.

## Results

### Abundant retrogenes in the zebrafish genome

**Figure 1:**
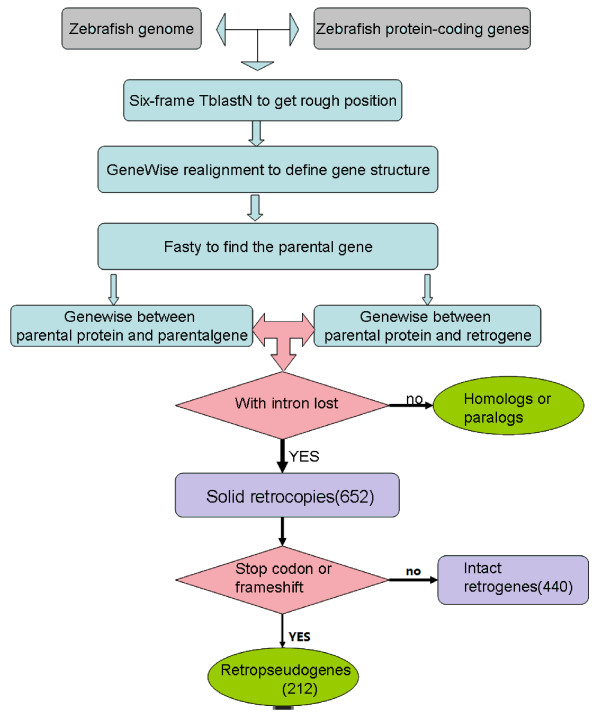
**Identification of retrocopies in the zebrafish genome**.

The 31743 protein sequences downloaded from the Ensembl database were used to detect retrogenes in the zebrafish genome with a computational pipeline with stringent parameters depicted in the flow chart shown in Figure 1. All 31743 protein sequences were mapped on the genome with TblastN [30], and 12504 longest regions mapped with the proteins were retained. Nearby homology matches (distance < 40 bp), which were not likely separated by introns, were merged after a series of Perl scripts' parsing. The structure of the 12504 sequences was obtained by GeneWise[31], and only 12333 sequences with intron larger than 40 bp were retained. To find the parental gene, the 12333 sequences were mapped with the whole 31743 protein sequences with FASTY[32], and we only kept the 8543 results that had the best score but no overlap with the protein position on the genome. Meanwhile the two sequences had more than 50% of the gene sequence's length and identified more than 50% on the amino acid level.

We only included the gene pairs of the Ensembl peptides with multiple coding exon (parental gene) as the best hit of the merged sequence (new gene), as these can prevent the single exon gene, e.g., olfactory receptor genes, from becoming the "parent" gene. Finally, if the sequence of the new gene overlapped two exons of the parental gene and the distance between the two exons was larger than 70 bp, we confirmed the new gene as a retrogene. We chose 70 bp as a threshold based on the following: 1) the majority of introns in zebrafish genome are larger than 70 bp [33], so it can exclude small gaps in parental genes annotated as introns by mistake; and 2) we ensured that at least one intron was lost in the new retrogene since 70 bp is larger than the gap size (40 bp) we used in the merging step. Ultimately, 6,021 retrogene candidates were found that satisfied all our criteria. To divide these candidates into primary retrogenes and duplicates that descend from primary retrogenes, we used the GeneWise [31] to determine whether the lost intron is derived from the parental gene. This produced 652 primary retrogenes, out of which 212 retrocopies with either frame-shift mutations or premature stop codons were defined as retropseudogenes. The rest of the retrogenes (440) were defined as intact retrogenes. (See Additional file 1). Actually, the number of primary retrogenes is most likely higher than this sample set, because some intronless copy could have originated through retroposition of old retrogenes.

**Figure 2:**
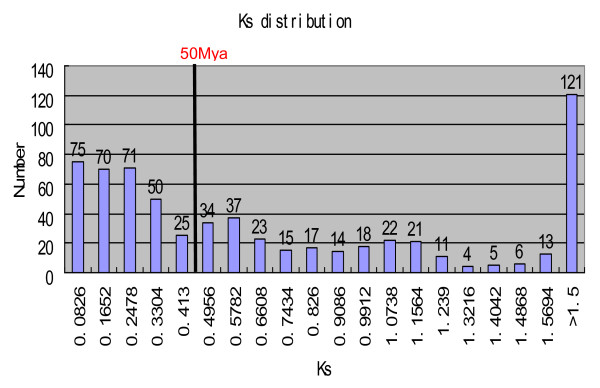
**Ka/Ks ratios in retrogene between the retrogenes and its parental sequences**.

To obtain the age distribution of all the retrocopy formation events, we plotted the Ks distribution of the parental-retrocopy pairs (Figure 2). Based on the divergence time of *Danio rerio *and *Cyprinus carpio *of 50 Mya (million years ago) [34], we used 38 pairs of orthologs between the two fishes, and found that the synonymous substitution rate of zebrafish genes is 4.13×10^-9 ^substitution per silent site per year. We found that the majority of retrocopies formed within the past 50 million years, indicating that recent, rapid formation of retrocopies may not have only occurred in primate lineages [29,35].

### Large amount of transcribed retroposed genes in the zebrafish genome

Generally, retrocopies were thought of as sequences without transcriptional ability, but many transcribed retrogenes were found in the primate and rodent genomes [11,15,26-29]. To explore whether this pattern exists in the zebrafish genome, we used ESTs to represent the transcription ability of these retrogenes, because they provide better discrimination within close paralogs than short-tag expression sequences or data from hybridization-based methods [36,37]. To map ESTs to the retrogenes, we used the rigorous pipeline of Vinckenbosch et al. (2006) that excludes erroneous mapping to parental genes or other paralogs (see methods). These analyses showed that about two-thirds of retrocopies (437 of 652 or 67%) matched with at least one EST, revealing that the majority of retrogenes are transcribed. (See Additional file 2) This proportion is much higher than that in human genome (1080 of 3590, or 30.1%). This might be because the retropseudogenes were about 84% of all the retrogenes (3015 of 3590, or 83.9%), but the expression of the retropseudogenes in human genome (26.8%) is much lower than in zebrafish genome (60%, see below)[38].

We investigated how the new retrogenes recruited new regulatory sequences. We fully screened the genomic context of retrogenes to explore the potential donors of the regulatory elements. We first compared the number of transcribed retrogenes located inside and outside of other genes. In the 440 intact retrogenes, we found that 198 were inserted into "host" genes and 242 between the two previous existing genes. Among the 198 retrogenes within a gene, 152 (76.7%) had at least one EST, suggesting they recruited the regulatory sequences from parental genes. Interestingly, this proportion of 76.7% was higher than the proportion of transcribed intergenic retrogenes (285 of 454, or 62.7%), indicating the retrogenes inserted inside a gene were more likely transcribed as a consequence of recruitment of a *cis*-regulatory element from the host gene.

For the retrogenes that were located outside a gene, we hypothesized that these retrogenes were transcribed because they were inserted into a genomic region that contained a transcriptionally more active region with more potentially active regulatory sequences. Such a retrosequence would be more easily to pick up regulatory elements. By contrast, if a retrosequence were inserted into a transcriptionally inactive genomic region with less potentially active regulatory sequences, then the retrogenes would be less likely to pick up a regulatory sequence and become processed pseudogenes. A prediction based on this hypothesis is that the regions flanking functional retrogenes would transcribe more RNAs; the regions flanking processed pseudogenes would transcribe less RNAs. We tested whether regions surrounding transcribed retrogenes are more active than regions surrounding transcribed silent retrogenes. We compared the number of ESTs mapped to the surrounding regions, in which transcribed retrogenes have more ESTs (median number of ESTs, 76) than transcribed silent retrogenes (median number of ESTs, 28), and found that the difference was statistically significant. (P < 10^-5^, Mann-Whitney U test).

### Most zebrafish retrogenes are very likely to be functional new genes

In the 652 primary zebrafish retrogenes, we only found 212 processed pseudogenes (32%), which contained frame shift mutations or premature stop codons. We further tested the functionality of the rest of the 440 (68%) retrogenes by using two approaches.

First, it is likely that a pseudogene without function may sometimes also be transcribed [39]. Hence, the previous test of transcription of intact retrogenes ought to be further examined to test their functionality. We compared the transcription patterns between the 440 retrogenes with intact ORF and 212 retropseudogenes. We found that the percentage of intact retrogenes with at least one EST (308 of 440 or 70%) was significantly higher than that of pseudogenes (129 of 212 or 60%) (P < 0.05, Fisher's exact test). The more significant pattern was observed (Table 1) when we used mRNA as transcription evidence (127 of 440 intact *vs *10 of 212 pseudogene, P < 10^-14^, Fisher's exact test). These data show that intact retropseudogenes are likely expressed and functional, compared to the hypothetical processed pseudogenes. It also should be noted here that the two criteria of disabled mutations, frameshift or nonsense mutations, to annotate a processed pseudogene may be too stringent, because it has been found that some functional genes can be formed by splicing out the gene regions that harbor frameshift and nonsense mutation as new introns [40,41].

**Table 1:** Retrogenes transcription supported by expression data

*Expressed info*	*Retrogene type(number)*	Number(percentage in this type)
mRNA	Intact(440)	127(28.86%)
	Pseudo(212)	10 (4.72%)
EST	Intact(440)	308(70.00%)
	Pseudo(212)	129(60.85%)

An overview of the highly transcribed retrogenes (as judged by the number of matching ESTs) also offers compelling evidence for transcription as a marker of retrogene functionality (Additional file 2). Among the 50 most highly transcribed retrogenes, the vast majority (39 of 50 or 78%) was intact, whereas only a small part (11 of 50 or 22%) was pseudogenes. A similar result was obtained in an extended analysis with the 100 most highly transcribed retrogenes in which 77 of 100 were intact retrogenes. Similarly, many retrogenes with a number of ESTs have been previously identified as functional genes.

Second, we investigated the sequence constraint created by functionality through a comparison of nonsynonymous(Ka) and synonymous(Ks) substitution rates (Ka/Ks ratios) between retrogenes and their parental genes. Generally, the evolution of pseudogenes is under neutrality, which means that the Ka/Ks values are larger than the ratio of genes subject to functional constraint under purifying selection, and smaller than the ratio of genes under positive selection [42]. This method has been widely used in identifying the functionality of genes [1,43,44]. We found that processed pseudogenes have much higher Ka/Ks values than intact retrogenes, illustrating that intact retrogenes are subjected to functional constraint. In the comparison between retrogenes with unknown constraints and parental genes that are known to be functional, a stricter criterion should be Ka/Ks < 0.5 to identify functionality of the retrogenes [11,15]. We found that a Ka/Ks ratio of 65% (284 cases) of intact retrogenes was significantly lower than 0.5. These data suggested that the intact retrogenes are under stronger purifying selection and are likely functional. The data also suggested that the processed pseudogenes are also subject to sequence constraint, showing that some of them are either functional in splicing out the disabled mutations or have been functional but recently became pseudogenes by disabled mutations.

### High proportion of chimerical genes

**Figure 3:**
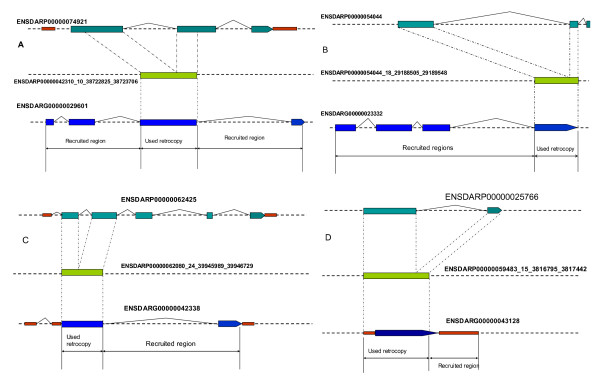
**Four examples of chimerical retrogenes**. A. both of the two terminuses was added a new peptide. B. new peptide was added to its N terminus. C. new peptide was added to its C terminus. D. part of the N terminus was change to 5'UTR. Limegreen box, used retrogenes. Teal box, parental CDS regions. Blue box, chimerical CDS region. Crimson box, UTR regions.

In contrast to rare recent chimerical retrogenes in mammalian genomes [12,45,46], zebrafish retrogenes recruit not only novel regulatory elements but also new coding sequences from the insertion sites and thus give birth to many chimerical genes that translate hybrid proteins. Out of the 440 intact zebrafish retrogenes, 95 (22%) were predicted to have chimerical protein coding sequence (CDS) structures (See Additional file 3). Among these cases, 26 retrogenes were added with a new peptide to the C terminus, another 26 retrogenes were added with a new peptide to the N terminus, whereas in 32 other retrogenes, both of the N and C terminus were added with a new peptide. For example, in the retrogene ENSDARP00000042310_10_38722825_38723706, both of the two termini were added with a new peptide, while in ENSDARP00000054044_18_29188505_29189548, a new peptide was added to its N terminus and in ENSDARP00000062080_24_39945989_39946729, a new peptide was added to its C terminus. Furthermore, in the retrogene ENSDARP00000059483_15_3816795_3817442, a part of the N terminus was changed to 5'UTR in the new hybrid protein structure (Figure 3).

All of the 84 retrogenes above were treated as coding fusion with neighboring genes. Whereas in the rest of the 11 retrogenes, 8 recruited 5' and 3'UTR to form a new single exon gene and 3 retrogenes cover the full length of the new chimerical gene, which were thought to be *de novo *chimerical genes. This pattern is much different from the pattern within the human genome, which has 36 fusion and 27 *de novo *chimerical retrogenes[38] (P < 0.01, chi-square test).

In the 95 hybrid new genes, 30 retrogenes overlap with the exon-intron boundary, which indicated that new splice sites were created during the evolutionary history of the retrogenes. A possible reason was that during evolution, new introns were produced in the original transcripts that included the retrogenes[47,48] or new splice sites were created from previous exon regions of the parental genes[41].

There was considerable expression evidence that some of the zebrafish hybrid genes might have acquired new protein functions. To further study the transcription of these hybrid genes, we used full-length mRNA and EST to blast against the transcript of the hybrid genes, and found that 73 hybrid genes have transcription evidence. Combined with the EST data, 26 chimerical retrogenes were among the top 100 transcribed retrogenes. The evidence above illustrated that most of the chimerical retrogenes were functional. Because of the extensive structural variation, these new chimerical genes were under the evolutionary process of neofunctionalization.

To explore the rate of creation of chimerical retrogenes, we counted the Ks distribution of the chimerical retrogenes that were under 1.5. Among the 62 retrogenes, ten of them had Ks values lower than 0.0826, which means 10 chimerical retrogenes formed in the last 10 million years, a rate that is 6 times faster than the rate of 0.14 chimerical retrogenes per million years in the evolution of the primate lineage toward humans [29,38]. If we extended the time to 100 million years, 52 chimerical genes were found and the rate, 0.52 genes per million years, is still about 4 times faster than that found in human. This is among the most rapid rates of generation of chimerical genes, just next to the rice [20].

To know the generation rate of chimerical retrogenes, we plotted the Ks distribution of retrogenes whose Ks was smaller than 1.5. Combined with the synonymous substitution rate of zebrafish genes, our results suggest that the formation of many chimerical retrogenes occurred at a remarkably rapid evolutionary rate, much faster than that of the primate lineage.

## Discussion

Retrogenes have been seen as evolutionary dead ends with little functional significance for a long time because of their low survival rate [49]. However, when analyzing the zebrafish genome, we found that retroposition was involved in producing a large number of new functional genes. In combining EST with full-length mRNA information, we found some of the new genes might have evolved new functionality during the evolutionary history after the insertion of retrogenes. We used the transcription information and values of Ka/Ks as indicators to testify to the functionality of these retrogenes, and found that a large proportion of retrogenes are transcribed, a finding that might indicate their functionality.

In this study, we have found that the transcription of retrogenes is not accidental by using a targeted approach. Our data indicate that retrogene transcription is very common and the transcriptional pattern of zebrafish has been profoundly influenced by natural selection. The finding of a large amount of retrogene transcription is consistent with the fact that a large number of retrogenes are functional [20,50]. Meanwhile, we found the regulatory sources for the transcriptional activity of retrogenes. Some of retrogenes seem to depend on the regulatory sequences of other genes, for example, by recruiting the regulatory sequences of neighboring gene or even directly fusing to host gene. Moreover, some retrogenes used the regulatory sequences of its own sequences. Thus, we predicted that the regions surrounding the position of the inserted retrogenes can influence the expression of the retrogene, and the chimerical retrogene itself can form new gene structure by recruiting new splicing sites.

The ratio of intact retrogenes is very different with that of the human genome, in which intact retrogenes are only a small proportion [38] and which are the result of a burst of young retrogenes in mammal genomes [29,35]. One reason may be that although there are many more kinds of LINE-1 in zebrafish than in human, but the total number of LINE-1 is less than that in the human genome [24,25]. Another reason might be that the evolutionary rate in teleostei is faster than in mammals [51,52], which makes the pseudogene inserts experience quick turnover (i.e., birth and death of retrotransposons). So, the retropseudogenes with frameshift mutations or premature stop codons are a smaller proportion in zebrafish than in the human genome [29].

Chimerical retrogenes have been reported in many species and are considered to be a very important component of protein diversity [53,54]. Recent studies have uncovered several young chimerical retrogene in primates (e.g. TRE2, TRIM5, PMCHL), in *Drosophila *(e.g. *jingwei, sphinx*) and in rice, which improves understanding of the molecular process of generating chimerical retrogenes. The systemic search of chimerical retrogenes in our study illustrates that their formation is driven not only by the merging of retrogene and by existing unrelated regions but also from inside the retrogene.

Our results suggest that many new chimerical retrogenes may have originated in zebrafish at a remarkably rapid evolutionary rate 6 times faster than the evolution of the primate lineage toward humans. This tentative estimate represents a lower bound for two reasons. First, we only searched the intact retrocopies for the chimerical genes, although new chimerical genes may have emerged from truncated coding regions [14]. It is also known that new splicing signals in a coding region that contains premature stop codons or frameshifts may evolve to form a new intron or to generate chimerical genes with "host" genes [14]. Second, duplicated "retropseudogenes" may play functional roles by means of their RNA regulating closely related paralogous genes [55,56].

## Conclusions

To examine the birth of these putative retrogenes, we have developed a stringent pipeline for the annotation of retrogenes and retropseudogenes in the zebrafish genome, from which we obtained 652 retrocopies. By using the EST and mRNA as expression evidence, we found that the majority of retrocopies were transcribed. Combined with the evolutionary analysis, we predicted that many retrocopies were functional genes. In addition, 95 retrogenes have recruited new exons or sequences from flanking regions; generating large numbers of chimerical genes, suggesting that gene origination through retroposition is ongoing, with a rate 6 times faster than the rate in humans. This indicates that the functional retrogenes have kept the zebrafish genome in constant flux. In addition retrogenes play an important role in the genome evolution and the retrotransposition provides a strong force for the adaption and speciation of the teleostei fish.

## Methods

### Defining the zebrafish retrogenes

For the zebrafish genome sequence, all annotated peptide sequences and expression data were downloaded from the Ensembl http://www.ensembl.org database (version: Ensembl 50). To define the zebrafish retrogenes, we used a method similar to the one used in identifying the retrogenes in the human genome [29]. To screen for retrogenes, the 31743 annotated protein sequences were used as queries to search against the whole genome sequence in translated similarity using TblastN [30] with an E-value threshold at 10-3. Adjacent homology matches were merged together using Perl scripts, combing only nearby matches (distance < 40 bp) that were not likely separated by introns. The merged target sequences and the query were thought as true if they, on the amino acid level, had significant similarity (identity > 50%) and overlapped with one another more than 70% of their sequence's length (at least 50 amino acids). GeneWise with default setting and a filtration score at 35 was then used for defining the intron-exon boundary of the merged target sequences. Next, FASTA [32]was used to perform similarity searches of merged target sequences against all Ensembl genes(intron-containing and intronless). We kept only copies in which the closest match was an Ensembl peptide with multiple coding exons (putative parental genes). Merged sequences for which the closest match was an intronless gene were excluded from the data (e.g. intronless gene such as olfactory genes). In this step, we also discarded retrocopies that originated from the duplication of other retrocopies. To confirm the absence of introns in these retrocopies, two GeneWise processes were carried out. First GeneWise was conducted between the protein sequence of putative parental gene and its genomic sequence, which provide all the intron position and length of putative parental gene. Second GeneWise was conducted between the protein sequence of the putative parental gene and its retrocopy (generally, there should not be any intron in this result). By manually checking the two results, we confirm the retrocopy as a real retrocopy if the introns in first result were lost in the second result. This produced 652 retrocopies. Out of the 652 retrocopies, 212 were defined as retropseudogenes with the occurrence of either frameshift mutations or premature stop codons. Ka and Ks substitution rates and Ka/Ks ratios were calculated using the Ka_Ks calculator program following the LPB methods[57] between the retrogenes and their intron-containing parental genes.

### Identification of chimerical retrogenes

In order to find chimerical retrogenes from all of the retrogenes, we first obtained the genome position of retrogenes and their parental genes as well as all the Ensembl transcripts using the BLAT program[58]. All of the position of the transcripts and retrogenes were obtained, and the retrogenes that overlapped with the transcripts were kept for further analysis. Second, we only kept the transcript that both overlapped with the retrogene and its parental gene for less than 80% of the length of their sequence, and we defined the transcript as a chimeric gene.

### Transcription analysis of the retrogenes

All of the ESTs downloaded from the University of California, Santa Cruz, database (assembly June 2009) were mapped to the zebrafish genome. The map result only kept the best BLAT hit of each EST as well as other hits that had an identity value in the nucleotide that falls less than 0.5% of the best hit and had at least 96% nucleotide identity with the genome sequence. To properly discriminate the transcription between the parental gene and retrogene, we proceeded as follows: 1)we only kept the ESTs mapped to a unique location on the genome matched in the University of California, Santa Cruz database criteria; that aligned with the genome sequence of >100 bp; and that had a nucleotide identity of >97%. 2) Among these ESTs, we only preserved the ESTs that produced an alignment with a genomic sequence that overlapped with a retrogene. In addition, we used ESTs as evidence to support multiexonic transcripts (retrogenes with gene fusions and new exons). We thought of an EST as confirmative evidence if it aligned both with a retrogene-derived and a non-retrogene-derived exon.

### Level of transcriptional activity surrounding retrogenes

Based on the BLAT results of all ESTs to the genome, we excluded ESTs mapped within the 2-kb flanking sequences of retrogenes, and counted the numbers of ESTs aligned with the retrogenes' 40-kb flanking sequences. We thought that the number obtained was an indicator of the level of transcriptional activity in zebrafish genomic regions surrounding retrogenes.

### Distance to closest gene

We obtained the position of the retrogenes and Ensembl transcripts (start and end) by BLAT and computed the minimal distance between a retrogene and its neighboring gene. We did not consider the orientations of the retrogenes and the Ensembl transcripts for this analysis (transcripts on the sense or anti-sense strands of the retrogenes were treated equally). Ensembl transcripts that overlapped with the annotated retrogenes were removed in this analysis.

## Authors' contributions

BF developed the algorithm, carried out the molecular genetic studies, performed the sequence alignment and drafted the manuscript. MC participated in algorithm development. MZ participated in the sequence alignment and the data analysis. ML participated in the design of the study. SH conceived of the study, participated in its design and coordination and helped to draft the manuscript. All authors read and approved the final manuscript.

## Supplementary Material

Additional file 1 **The 652 retrogenes identified in this study**. Click here for file

Additional file 2 **The 437 retrocopies with at least one EST supported**. Click here for file

Additional file 3 **The 95 chimerical retrogenes identified in this study**. Click here for file
